# Inhibitory Pathways for Processing the Temporal Structure of Sensory Signals in the Insect Brain

**DOI:** 10.3389/fpsyg.2018.01517

**Published:** 2018-08-21

**Authors:** Hiroyuki Ai, Ajayrama Kumaraswamy, Tsunehiko Kohashi, Hidetoshi Ikeno, Thomas Wachtler

**Affiliations:** ^1^Department of Earth System Science, Fukuoka University, Fukuoka, Japan; ^2^Department of Biology II, Ludwig-Maximilians-Universität München, Martinsried, Germany; ^3^Neuroscience Institute, Division of Biological Science, Graduate School of Science, Nagoya University, Nagoya, Japan; ^4^School of Human Science and Environment, University of Hyogo, Himeji, Japan

**Keywords:** cricket, disinhibition, duration coding, honeybee, moth, postinhibitory rebound, temporal structure, waggle dance

## Abstract

Insects have acquired excellent sensory information processing abilities in the process of evolution. In addition, insects have developed communication schemes based on the temporal patterns of specific sensory signals. For instance, male moths approach a female by detecting the spatiotemporal pattern of a pheromone plume released by the female. Male crickets attract a conspecific female as a mating partner using calling songs with species-specific temporal patterns. The dance communication of honeybees relies on a unique temporal pattern of vibration caused by wingbeats during the dance. Underlying these behaviors, neural circuits involving inhibitory connections play a critical common role in processing the exact timing of the signals in the primary sensory centers of the brain. Here, we discuss common mechanisms for processing the temporal patterns of sensory signals in the insect brain.

## Introduction

The temporal patterns of sensory signals can serve as critical cues in behavioral choice. Insects offer a striking advantage over vertebrates for analyzing morphology and physiology of neural circuits at the levels of single identified neurons (Menzel, 2006), so that the network architecture underlying cognitive function can be investigated in detail. Recently, the neurophysiological mechanisms for processing the temporal structure of sensory signals have been revealed in different sensory modalities in various insect species. These results not only suggest that the timed interaction of excitation and inhibition plays key roles in temporal pattern recognition in insects, but also unveil network architectures underlying the coding of sensory temporal structure. Here, we review temporal cognition and its underlying neural mechanisms in the olfactory system of moths and in the auditory systems of crickets and honeybees.

## Moth Odor Plume Following

**FIGURE 1:**
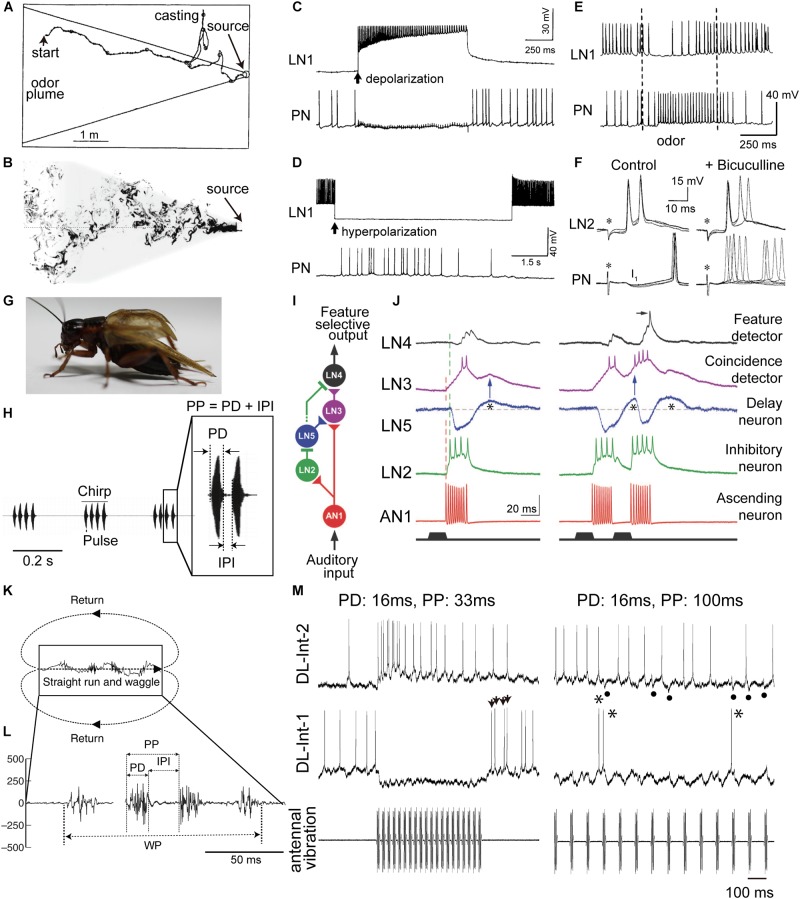
Insect communication signals and physiologies of critical interneurons involved in temporal processing of sensory signals. **(A)** Simulated flight path of a moth (winding curve) in a pheromone plume (area enclosed by solid straight lines). Repetitive exposure to sex pheromones in the plume is necessary for sustained upwind flight (from left to right) toward a pheromone source. When the animals lose the pheromonal stimulus, they cease to make upwind progress and instead begin casting. **(B)** The structure of a plume in turbulent flow from right to left. The shaded area represents the projection of the conical average plume. **(C–F)** Responses of key neurons in the moth odor processing circuit (see **Figure 2A** for a circuit diagram). **(C)** Depolarization-induced firing in the local interneuron 1 (LN1, top) led to hyperpolarization and suppression of firing in the projection neuron (PN, bottom). The PN suppression closely followed the onset of current injection in the LN1 (arrow), and spiking in the PN resumed immediately upon repolarization of the LN1. **(D)** Hyperpolarizing current injected into an LN1 caused an abrupt suppression of firing of the LN1, and this resulted in depolarization and firing in the PN. This firing in the PN occurred only during LN1 hyperpolarization. **(E)** Intracellular records from the LN1 and the PN during sex pheromone stimulation (between the two dashed lines). **(F)** Intracellular records from a local interneuron 2 (LN2, top) and a PN (bottom) responding to brief electrical stimulation of the ipsilateral antennal nerve (asterisks, left). PNs show fast inhibitory postsynaptic potentials (IPSPs; I_1_ shown left of PN), which disappeared when the gamma-aminobutyric acid (GABA)-receptor blocker bicuculline was applied, resulting in increased variability in the timing of evoked spikes (right of PN). **(G)** Male crickets produce a calling song by rubbing both forewings together. **(H)** Audio signal of the calling song in the Mediterranean field cricket (*Gryllus bimaculatus*). Females are selectively attracted to the pulse pattern of the conspecific calling song. Each chirp has a temporal structure with a fixed pulse period (PP), consisting of pulse duration (PD) and interpulse interval (IPI). **(I)** Neural network for detecting the temporal (structure of the male cricket calling song. **(J)** Intracellular membrane potential records of critical interneurons in cricket auditory processing. A postinhibitory rebound (PIR, indicated by asterisks) excitation plays a critical role in song detection. **(K)** Moving trajectory of a honeybee during the waggle dance. The dance consists of a waggle phase (WP) and a return phase. The distance to the flower source is encoded as the duration of the WP of the dance. **(L)** Thoracic vibration velocities recorded during the WP. Intermittent vibration pulses occur with a constant PD of about 16 ms and a PP of about 33 ms. **(M)** Intracellular records of dorsal lobe interneurons 1 (DL-Int-1, middle) and 2 (DL-Int-2, top) in the primary auditory center of the honeybee (see **Figure 2E** for a circuit diagram) in response to vibratory mechanical stimulation to an antenna (bottom). Left: When the PPs are shorter than 50 ms, the DL-Int-1 receives strong inhibition that allows no spikes during the pulse trains and exhibits a PIR excitation (arrowheads) upon the offset of the pulse train. DL-Int-2 exhibits elevated spiking activity during stimulation. Right: DL-Int-1 shows spikes (asterisks) intermittently during the IPI phase when the PP of the stimulus is longer than 50 ms. Under this condition, the DL-Int-2 often shows a lack of spikes with remarkable IPSPs (dots). Modified from Kaissling (1997) for **A**; Celani et al. (2014) for **B**; Christensen et al. (1993) for **C**, **D**, and **E**; Christensen et al. (1998) for **F**; Hedwig (2016) for **H**; Schöneich et al. (2015) for **I** and **J**; Hrncir et al. (2011) for **K** and **L**, with the permission of Birkhäuser Verlag for **A**; Springer Nature for **C**, **D**, and **E**; The Society for Neuroscience for **F**; The American Association for the Advancement of Science (AAAS) for **I** and **J**; and The Company of Biologists for **K** and **L**.)

**FIGURE 2:**
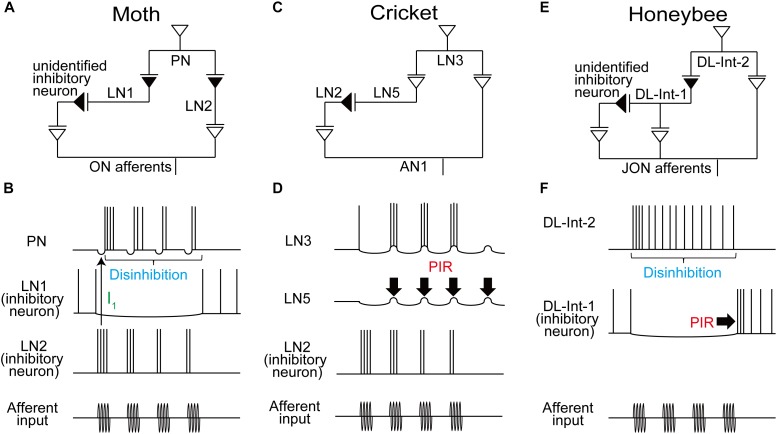
Comparison of the neural circuits and response patterns of the moth **(A,B)**, cricket **(C,D)**, and honeybee **(E,F)**. **A**, **C**, and **E**: putative neural circuits for processing the temporal structure of sensory signals. **B**, **D**, and **F**: response of each neuron in these circuits. Disinhibition is found in the moth from LN1 to PN **(B)** and in the honeybee from DL-Int-1 to DL-Int-2 **(F)**. In the moth, LN2 induces a fast IPSP (I_1_; thin arrow) for phase-locking the timing of the PN spikes. PIR (thick arrows) excitation may plays a critical role in detecting cricket song IPIs **(D)** and also in detecting the end of the waggle phase in the honeybee **(F)**.

Many insects orient themselves toward conspecifics or food sources using odor cues. As a model system of this behavior, the sex pheromone response of the male moth has been studied extensively. The male moth is attracted by sex pheromones and approaches the odor source, the female. While approaching the source, the animal changes its flight course if it detects the odor plume (**Figure 1A**; Kaissling, 1997). In turbulent air flow, the odor plume forms a cloud of unevenly distributed odor filaments entangled with non-odorized air pockets (**Figure 1B**; Celani et al., 2014). Therefore, the sensory organs that detect the odor molecules, the antennae, experience intermittent odor pulses. Consequently, as the animal approaches the odor source, it receives the odor pulses in short intervals. Such intermittent detection of sex pheromones is indeed essential for sustained upwind flight (**Figure 1A**; Baker et al., 1985) or walking (Kanzaki, 1997) toward a pheromone source. Flying moths cease to make upwind progress and begin to “cast” across the wind line when they lose the pheromonal stimulus, that is, when the intervals between odor pulses become longer than those in the odor cloud (Baker et al., 1985). The neural circuit for encoding the temporal structure of odor stimuli has been clarified (Christensen et al., 1993, 1998). In the primary olfactory center of the moth antennal lobe, most local interneurons (LNs) use an inhibitory neurotransmitter, gamma-aminobutyric acid (GABA), and two types of LNs (named LN1 and LN2 in this review) and projection neurons (PNs) are involved in olfactory signal processing (see **Figure 2A** for a circuit diagram). Indeed, current-induced firing in the LN1 led to hyperpolarization and suppressed firing in the PN (**Figure 1C**). The time course of the PN suppression closely followed the period of current injection in the LN1, and spiking in the PN resumed immediately upon repolarization of the LN1 (**Figure 1C**). Conversely, hyperpolarizing current injected into another LN1 caused an abrupt suppression of firing of the cell, and this resulted in depolarization and firing in the PN (**Figure 1D**). This firing in the PN occurred only during LN1 hyperpolarization. This relationship between LN1 and PN also occurred during sensory stimulation. When the antenna was exposed to the principal sex-pheromone component, Bombykal, the LN1 was inhibited, and firing was suppressed (**Figure 1E**). The decreased firing in the LN1 was associated with increased firing in the PN. The period of elevated PN activity approximated the duration of the odor pulses. These results suggest that the firing of the PN is allowed by a disinhibition (inhibition of an inhibitory neuron) of PN1, and that suppression of the inhibitory LN1 is the mechanism underlying the disinhibition (**Figures 2A,B**; Christensen et al., 1993).

Another type of LN, named LN2 here, receives excitatory input from olfactory sensory afferents (Christensen et al., 1998). Since LN2s are inhibitory neurons, their postsynaptic neurons, PNs, show fast inhibitory postsynaptic potentials (IPSPs; I_1_ in **Figure 1F**) in response to stimulation of the ipsilateral antenna. IPSP responses of PNs disappeared when the GABA receptor blocker bicuculline was applied, resulting in increased variability in the timing of evoked spikes (**Figure 1F**). When short intermittent pulses of female sex pheromone were applied to the antenna, as in the odor plume, the moth advanced directly toward the odor source, and each stimulus pulse evoked a train of spikes in the PN that was linked to the intermittent stimulus pattern (**Figure 2B**; Christensen et al., 1998).

Thus, the moth uses inhibitory pathways for detecting the timing of both the onset, through LN2, and the continuation, through LN1, of odor pulses (**Figures 2A,B**). A simulation study of the neuronal circuit that processes the temporal structure of olfactory stimuli in moths also indicated that individual PNs, but not individual olfactory receptor neurons (ORNs), encode the onset and offset of odor puffs for any temporal structure of stimuli (Jacob et al., 2017).

## Cricket Calling Songs

Temporal signal processing mechanisms are used not only in olfaction but also in audition. Male crickets produce sound pulses by rubbing their forewings together (**Figure 1G**). Each pulse has a carrier frequency around 5 kHz, and repetitive pulses constitute a chirp (**Figure 1H**; Huber et al., 1989). The temporal structure of male songs is species-specific and is used to attract conspecific females for successful mating. The interpulse interval (IPI) is one of the key parameters underlying this behavior (Hedwig, 2006). IPIs are rather fixed, which is favorable for experimental manipulation and analysis. A recent study by Schöneich et al. (2015) clarified the mechanisms underlying such IPI-selective responses in the Mediterranean field cricket (*Gryllus bimaculatus*). A chirp of this species consists of 3–4 repetitive sound pulses, with a pulse duration (PD) of 15–23 ms and an IPI of 16–24 ms (**Figure 1H**). Results of electrophysiological experiments suggest the following neural pathway for IPI selection (**Figure 1I**; Schöneich et al., 2015): The male song is received by the tympanic auditory organ, located on the forelegs in females. The sensory afferents of the organ project to an auditory neuropil in the prothoracic ganglion, in which an ascending neuron 1 (AN1) has its dendritic arbor (Wohlers and Huber, 1982). AN1 encodes temporal patterns of the song in its spike trains. While AN1 has a direct excitatory synapse on an excitatory local interneuron 3 (LN3) in the brain, AN1 also indirectly excites LN3 via an inhibitory LN2 and an excitatory LN5. LN2 is directly activated by AN1 during a sound pulse and evokes a lasting hyperpolarization, i.e., inhibition, in its postsynaptic LN5. This inhibition gives rise to a rebound depolarization of the membrane potential (**Figure 1J**, asterisks), a so-called postinhibitory rebound (PIR). LN5 is a graded-potential neuron that does not exhibit action potentials but will release neurotransmitters depending on membrane depolarization; LN5 thus excites its postsynaptic LN3 upon PIR (**Figure 1J**, blue arrows). Because of this circuit organization, LN3 is excited most strongly by pulses with an appropriate IPI in which the delayed excitation from the PIR in LN5 upon a pulse arrives coincidentally with the direct excitation from AN1 evoked by the subsequent pulse (**Figure 1J**, right). Thus, LN3 functions as a coincidence detector that shows selective response to certain IPIs. These results suggest that crickets use a combination of a coincidence detector function and a PIR-based delay mechanism for detecting the timing of pulse patterns in auditory communication signals (**Figures 2C,D**).

Notably, the PIR occurs at a fixed delay of about 40 ms from the end of each pulse, and this corresponds to the pulse period (PP), the sum of PD and IPI. This observation accounts well for the preference of the LN3 response to the overall pulse structure of conspecific songs; LN3 also responds selectively to PP in addition to IPI (Kostarakos and Hedwig, 2012). Finally, LN4 is also suggested to receive inhibitory inputs from LN2 (**Figure 1I**). Therefore, upon the first stimulus pulse, LN4 cannot evoke spikes even if excitatory input is received from LN3 (**Figure 1J**, left). However, upon the second pulse, LN4 receives a stronger excitation from LN3 due to the PIR-based excitation from LN5, resulting in an overshoot evoking spikes (**Figure 1J**, right). Thus, LN4 can function as a temporal feature detector that shows even sharper selectivity for the combination of conspecific IPI and PP.

## Honeybee Waggle Dance

Honeybees convey the spatial information of profitable flower sources to hive mates using their waggle dance, in which the duration of the waggle phase (WP) increases proportionally with the distance to the flower source (von Frisch, 1967). During the WP, the dancers vigorously shake their abdomens while beating their wings at about 265 Hz (**Figure 1K**). The other individuals follow the dancer’s abdomen, receiving intermittent vibration pulses caused by the dancer’s wing beats. The pulses have a constant PD of around 16 ms and a PP of around 33 ms (**Figure 1L**). The airborne vibrations are detected by the vibration-sensitive sensory organs in the antennae, called Johnston’s organs (JO; Dreller and Kirchner, 1993), and the vibration signals are transmitted to the primary auditory center including the dorsal lobe (DL; Ai et al., 2007; Brockmann and Robinson, 2007). Anatomical and physiological evidence suggests a neural circuit for processing vibration pulses in the honeybee brain (Ai et al., 2017; **Figures 1M, 2E**). An identified DL neuron, DL-Int-1, is a GABAergic inhibitory neuron (Ai et al., 2017). DL-Int-1 shows spontaneous activity, but when trains of pulses with short pulse period (short PP) are applied to the antenna, DL-Int-1 shows remarkable hyperpolarization and the spontaneous spikes disappear (**Figure 1M**, left column). The mechanism of this inhibition of DL-Int-1 is still unknown. A PIR excitation (**Figure 1M**, arrowheads) appeared upon the offset of the pulse train. Under this stimulus condition, DL-Int-2, a presumed postsynaptic neuron of DL-Int-1, evokes continuous spikes (**Figure 1M**, top left). In contrast, when trains of pulses with long-PP are applied to the antenna, DL-Int-1 shows intermittent spikes during the train of pulses (**Figure 1M**, right column, asterisks), and DL-Int-2 often shows a lack of spikes with remarkable IPSPs (**Figure 1M**, dots). A computational analysis based on these data suggests that the honeybee may use a disinhibitory network to encode the duration of the WP: DL-Int-2 spiking upon excitatory input from JO afferents is elicited by an inhibition of the presynaptic inhibitory neuron DL-Int-1 (**Figures 2E,F**; Kumaraswamy et al., 2017). Importantly, DL-Int-2 spikes in response to stimulation by trains of pulses with short PP (**Figure 1M**, left), presumably as a result of the short-PP selectivity of the inhibition from DL-Int-1. Thus, the disinhibitory network contributes to the coding of not just the WP, but also the short PP. These experimental and computational results suggest the following motif that resembles the functions of a stopwatch: When a train of vibration pulse stimuli is applied to the JO, DL-Int-1 stops the spontaneous spikes via hyperpolarization. This termination of spontaneous spikes of DL-Int-1 could lead to the timing of spike burst onsets in DL-Int-2, like the start signal in a stopwatch. During the train of vibration pulse stimuli, tonic hyperpolarization of DL-Int-1 could sustain the spike burst on DL-Int-2 as long as the PPs are within the appropriate range, corresponding to a running stopwatch. When the vibration pulses stop, DL-Int-1 shows a PIR excitation, which inhibits the spike burst of DL-Int-2, like the stop signal in a stopwatch. Interestingly, sustained inhibition of a critical auditory neuron plays an important role in the selectivity for stimulus duration not only in insects, but also in anurans (Alluri et al., 2016). These findings suggest a common function across sustained inhibition in various species.

In addition to the airborne vibration caused by wingbeats, tactile contact of a follower’s antenna with the dancer’s body may also function as a signal related to the WP (Dyer, 2002; Michelsen, 2003; Gil and De Marco, 2010). The tactile contact deflects the antenna, which may be detected by neurons in the antennal joint hair sensilla. These sensory afferents also project to the DL (Ai et al., 2007), implying that the identified DL interneurons discussed here might also be involved in the processing of the temporal structure of the tactile contacts.

## Discussion

In this review, we compared the processing of the temporal structure of sensory signals in different modalities and different insect species. We particularly focused on the roles of inhibitory interneurons in determining the spike timing of postsynaptic neurons and thereby contributing to extracting temporal features. We highlighted two common characteristics, disinhibition and PIR, found across temporal processing circuits.

In the olfactory processing in moths and in the vibration processing of waggle dance signals in honeybees, a disinhibition is suggested to contribute to detecting the total duration of sensory stimulation (**Figures 2B,F**). In the anuran auditory system, a disinhibitory circuit motif was also proposed for counting the number of sound pulses that occur with a species-specific IPI (Naud et al., 2015). Thus, disinhibition might be a common mechanism across the animal kingdom for encoding the total duration of sensory input, which is equivalent to the product of the number of pulses with a fixed PP. PIR can serve to process IPIs in the cricket song (**Figure 2D**) and the offset of the WP in honeybees (**Figure 2F**). PIR also occurs in the mammalian auditory system, underlying selectivity for periodic low frequency amplitude modulations of sound signals (Felix et al., 2011) and, at a different time scale, spatial selectivity for sound location (Beiderbeck et al., 2018). Among the three insect models we reviewed here, the temporal processing circuits of the honeybee waggle dance employ both disinhibition and PIR (**Figure 2F**), allowing reliable encoding of WP; disinhibition results in a sustained response of the output neuron, DL-Int-2, to continuously detect waggling, and PIR excitation results in a signaling stimulus offset in the same neuron.

Inhibitory interneurons in insects have been suggested to be involved in various other aspects of sensory processing such as gain control (Olsen and Wilson, 2008), lateral inhibition (Sachse and Galizia, 2002; Silbering and Galizia, 2007), synchronization of spikes (MacLeod and Laurent, 1996; Bazhenov et al., 2001), and encoding of temporal stimulus patterns (Christensen et al., 1993, 1998; Hedwig, 2006, 2016). Here, we also explored the similarities between the neural processing of honeybee waggle dance signals and the neural processing of cricket audition and moth olfaction. Inhibitory inputs are also suggested to be critical in temporal processing in vertebrates. Future experiments should further elucidate the role of postsynaptic inhibition in encoding temporal signaling and in decoding the distance to a flower source in the foraging flight of the honeybee.

## Author Contributions

All authors listed have made a substantial, direct and intellectual contribution to the work, and approved it for publication.

## Conflict of Interest Statement

The authors declare that the research was conducted in the absence of any commercial or financial relationships that could be construed as a potential conflict of interest.
